# A survey exploring awareness and experience of tinnitus in young adults

**DOI:** 10.4102/sajcd.v64i1.545

**Published:** 2017-11-17

**Authors:** Vedika Bagwandin, Lavanithum Joseph

**Affiliations:** 1 Discipline of Audiology, University of KwaZulu-Natal, South Africa

## Abstract

**Background:**

Hearing loss among young adults is on the increase largely because of greater exposure to recreational noise. One of the effects of a sensorineural hearing loss is tinnitus. Despite efforts to raise awareness of hearing loss and tinnitus, young adults continue to expose themselves to the potential risks. The reasons for this are unclear; neither is the extent to which tinnitus is experienced in this population.

**Objectives:**

The study aimed to describe the awareness and experience of tinnitus among young adults, with regard to its existence, causes, effects and management.

**Methods:**

A descriptive study design was employed using an electronic survey that targeted students within a selected school at a university in KwaZulu-Natal. There were 75 participants between 18 years and 30 years. Data were analysed using descriptive statistics. To raise awareness about tinnitus, on completion of the questionnaire, all participants were given access to an information document about tinnitus, its causes and management.

**Results:**

The majority of participants (69.86%) were not aware of the existence of tinnitus. Thus, most of the participants did not know about the causes or effects of tinnitus. Tinnitus was experienced by 13.51% of participants themselves and 12.16% knew someone who suffered from it.

**Conclusions:**

The general lack of awareness of what tinnitus was, its causes and effects, has implications for audiologists who are involved in hearing health care across the age span. Hearing health promotion programmes targeted at young adults should include information on hearing loss as well as tinnitus and its management.

## Introduction

Tinnitus is defined as a sound perceived in the ears and/or head without an external sound source (Alam et al., 2012). Its intrusive nature can interfere with the lifestyle of the affected individual, irrespective of age, gender, race and socio-economic status (Henry, Dennis & Schechter, 2005). Traditionally, studies on tinnitus have focused predominantly on older adults. However, recent studies have shown that approximately 18% of young adults between the ages of 18 and 25 experience tinnitus (Degeest, Corthals, Vinck & Keppler, 2014). The causes of tinnitus can include illness, certain medication, head and neck injury, surgery and exposure to excessive noise experienced at places such as nightclubs, raves and concerts (Nondahl et al., 2010). Some studies have found that chronic noise exposure has been reported as the main cause of tinnitus in young adults because of exposure to excessively loud noise from personal listening devices (PLDs), recreational activities or attending nightclubs (Gilles, Van Hal, De Ridder, Wouters & Van de Heyning, 2013; Salvago et al., 2012). However, it is important to note that there may be other confounding variables with regard to the population group in the current study when compared to the same population group of other studies. The burden of diseases including tuberculosis and ototoxicity associated with tinnitus, low socio-economic status and lifestyle influencing access to PLDs and nightclubs may be different in the South African young adult population.

It is evident though that young adults expose themselves to loud noise while participating in leisure activities (Degeest et al., 2014). It is believed that these young adults may listen to excessively loud music for reasons associated with social norms and expectations (Levey, Fligor, Ginocchi & Kagimbi, 2012). Thus, these individuals are at risk of acquiring tinnitus during recreational activities and by using PLDs and other sources of loud music (Vogel, Looij-Jansen, Mieloo, Burdof & de Waart, 2014). Therefore, it has been suggested that young adults should be educated about the harmful effects of excessive noise exposure in nightclubs, raves and concerts and about the advantages of using hearing protection devices (HPDs) (Gilles et al., 2013). However, it appears that despite the increasing prevalence of tinnitus and hearing loss in the young–adult population (Chung, Roches, Catherine, Meunier & Eavey, 2005), there is still a lack of knowledge about the impact of excessive noise on the ears and use of HPDs. Generally, hearing loss is associated with the elderly; therefore, it is unlikely that young adults may pursue the use of HPDs. Thus, it may be beneficial to strive to change the attitudes of young adults (Gilles et al., 2013).

As in older adults, young adults may also experience the negative effects of tinnitus in their daily lives. Some of the most common effects of tinnitus reported in young adults include difficulty falling asleep, feeling depressed, irritability, interference in social activities, hearing difficulty, feeling confused and experience of disruption of daily activities and jobs (Zeman, Koller, Langguth & Landgrebe, 2014). In fact, these unwanted effects may be more detrimental to the young adult in that they may affect their academic studies. It is assumed that these negative effects of tinnitus may cause an increase in the failure and dropout rates at learning institutions and also prevent them from obtaining employment. According to the World Health Organization, health is described as total mental and physical fitness which allows an individual to participate in daily life (WHO, 2007). If young adults experience the unwanted effects of tinnitus such as trouble falling asleep, concentration difficulties and psychological complications, this may negatively impact on their daily life including social interactions and academic and vocational progression. Some studies have suggested that there may be a relationship between tinnitus, anxiety, depression and suicide in young adults who suffer with tinnitus (Cho, Chi, Song, Lee & Kim, 2013). Researchers revealed that 10% of 973 young adults from a Dutch inner-city senior-secondary vocational school, who participated in their study, reported depression and suicidal thoughts after experiencing tinnitus and hearing loss and thus sought help (Vogel et al., 2014).

Although there are various methods of managing tinnitus, such as the use of hearing aids, counselling, acoustic therapy, relaxation techniques, pharmacological and surgical approaches (Sweeto, 2013), young adults may not always be aware that there are management strategies for tinnitus and as a result may not seek help. It is also suggested that because young adults do not consider tinnitus as an important health problem, as there is no associated pain, they may not seek any help for it (Roberts, Hal Martin & Bosnyak, 2011).

It is clear that more awareness on what tinnitus is, its causes and effects has to be provided to young adults. However, it may be more beneficial to inform all young adults about the effects of tinnitus on their lives specifically rather than focus on how hearing loss and tinnitus occur generally (Roberts et al., 2011). If young adults are aware of how this affects them personally, they may be more likely to take precautions. This point of view is also promoted by other researchers who state that young adults are likely to perceive and understand risks involved with exposing themselves to detrimental situations only if it emerges from their own personal experiences (Landälv, Malmström & Widen, 2013). Tinnitus can manifest in various ways because of the underlying cause; and thus management would also differ depending on the reason for the tinnitus. While many tinnitus management strategies exist, young adults, in general, may not seek help because of their lack of knowledge regarding tinnitus management. This highlights the need for young adults to be better informed about tinnitus. It is also important that audiologists who provide services to tinnitus sufferers have knowledge about the extent to which this condition exists and manifests in young adults. Therefore, the aim of this study was to describe young adults’ awareness and experience of tinnitus.

## Research method and design

### Aim

The aim of the study was to describe the awareness and experience of young adults regarding tinnitus. This led to the following objectives:

- to describe awareness regarding the causes of tinnitus and its effects
- to describe experience and management of tinnitus.

### Design

A quantitative descriptive survey design was used as it aims to report on a large population and also serves as a means of describing attitudes, beliefs and opinions of individuals (Leedy & Ormrod, 2013). The data were obtained by means of a self-administered questionnaire that was distributed electronically. An electronic survey was chosen as it was a cost-effective way to obtain responses. It also facilitated data collection by allowing the researcher to obtain several responses in a short time (Hunter, Corcoran, Leeder & Phelps, 2013). It was also considered a viable method to communicate with this sample population as university students are expected to use electronic communication, including online learning at this university with access to computer laboratories.

### Sampling method

A non-probability purposive sampling method was used. Participants were selected according to their age range and the specific school in which they were registered at the university (Levey & Lemeshow, 2013). The participants were selected because they were easily accessible to the researcher (Leedy & Ormrod, 2013) while meeting the age criteria of young adults. The sample of participants comprised all undergraduate and postgraduate students in 2013, who met the inclusion criteria, from one non-health sciences school at the University of KwaZulu-Natal. This school had the most students while allowing for a level of homogeneity in the sample.

### Participants

Because this study focused only on young adults, the age group 18 years – 30 years was chosen. Some other studies, which focused on young adults at universities, also used participants within this age group as it best represented a young adult group (Agrawal, Platz & Niparko, 2008; Mostafapour, Lahargoue & Gates, 2009). The researcher chose university students specifically, as they would fit this age group easily.

### Selection criteria

Participants had to be between the ages of 18 years and 30 years and currently registered as students in the selected school. The participants needed to be undergraduate or postgraduate students in 2013. The researcher included the postgraduate students for two reasons. Firstly, the study required young adults from 18 years to 30 years and it is challenging to obtain participants from the 30-year age group who are undergraduate students. Secondly, there are many students who may have chosen to do postgraduate studies shortly after completing an undergraduate degree. These students would have been below the 30-year age group.

### Description of participants

Only 103 participants of a potential 4179 students responded to the survey. However, 75 completed the questionnaires. Only 74 (98.6%) participants confirmed their gender, of which 24 (32.43%) were males and 50 (67.57%) were females.

### Data collection instrument

The data collection instrument was developed by the researcher based on previous literature. The questionnaire used in the current study was a structured, self-administered questionnaire. The questionnaire comprised 32 questions with a range of closed-ended, open-ended, single-response and Likert-scale questions which probed various areas relating to the participants’ awareness of what tinnitus was, the causes and effects, as well as their experience of tinnitus. Closed-ended questions gave participants a choice to explain responses categorised as ‘other’. The questions provided an opportunity to express their experience of tinnitus in an easy, yet comprehensive, manner and also provided the researcher with information to achieve the aim and objectives of the study (Refer to Table 1 for a description of the questionnaire developed by the researcher). The questionnaire was adapted for online format for Survey Monkey software.

**TABLE 1:** Description of the questionnaire.

Section	Questions	Motivation
A	Biographical details	Participants were required to provide their age and gender. This information had an impact on the inclusion criteria of the study.
B	Awareness of tinnitus	These questions described whether the participants were aware that tinnitus existed, where they obtained this knowledge from and also if they had knowledge about the causes of tinnitus. There were six questions that focused on noise exposure, based on the literature which stated that noise exposure may be the key cause of tinnitus in this population group (Chung et al., 2005). Questions regarding illness, medication, injury and ear infections were included so that the questionnaire was not biased towards only noise exposure.
C	Experience of tinnitus	The questions included in this section focused on either the participant’s experience of tinnitus or that of someone else they knew. Obtaining this information fulfilled the second objective of the study as well as the main aim that tinnitus is being experienced at a much younger age (Eggermont, 2012). Questions on types of tinnitus as well as management of it served to determine whether young adults had access to management. It also served to find out whether their experience of tinnitus caused anxiety, fear, social and/or academic difficulties (Erlandsson, Holmes, Widen & Bohlin, 2008). Questions investigated the type, characteristics and possible causes of the tinnitus. It also focused on the treatment of tinnitus as well as the effects that tinnitus had on their life, which had implications for its management.

Refer to the Online Appendix 1 for the questionnaire used in the study.

## Procedure

### Reliability and validity

A pilot study was conducted to ensure that the questionnaire was clear and that participants had understood all questions. The pilot study was conducted on students in the same age group who were registered in another school at the university. It provided feedback clarity on the reliability of the electronic survey and assisted in determining whether the questions were appropriate, unbiased and provided an idea of possible analysis of the results. The questionnaire used simple and unambiguous language so that participants provided answers which focused on the response intended and related to the aim and objectives of the study in terms of face and content validity (Leedy & Ormrod, 2013). Clear instructions were provided to make it easier for participants to answer the questions.

### Data analysis

Data were analysed by inputting the results on the SPSS program, version 21 (IBM core). Descriptive statistical procedures such as frequency and percentages were used based on the type of questions being addressed. Results for awareness and knowledge, experience of tinnitus and noise exposure were analysed by using frequency counts and percentages. Mean average scores were used to analyse the average number of responses for the causes of tinnitus.

## Results

### The awareness of the existence of tinnitus

The results showed the majority of the participants in this study were not aware of what tinnitus was, as 51 (69.86%) stated that they had not heard of the condition. When participants were asked about their source of information on tinnitus, only 25 participants chose to answer that question. Thirty-two per cent of these 25 participants knew about the condition from friends and family who experienced it and from the Internet. Only one respondent reported being aware of the condition from their own experience. These results show that tinnitus is not commonly known and experienced and therefore participants were not likely to actively search for information on it.

### Awareness of the effects of tinnitus

Only two out of 74 participants who answered this question (2.79%) were aware about the effects of tinnitus. From these two participants, one reported that individuals might have ringing in the ears and decreased hearing for environmental sounds as effects of the tinnitus. The second participant did not specify what they believed to be the effects of tinnitus.

### Awareness of the causes of tinnitus

Participants were given a set of choices for the possible causes of tinnitus, each on a five-point Likert-scale, ranging from strongly disagree to strongly agree. Most of the participants (64.39%) agreed that PLDs cause tinnitus and another 61.81% agreed that ear-related problems cause tinnitus (Refer to Table 2 for awareness of the causes of tinnitus). Interestingly, it was also noted that participants viewed PLDs as a bigger cause of tinnitus than attending nightclubs, raves and concerts.

**TABLE 2:** Views about the causes of tinnitus.

Cause	Participants who agreed on the cause (scores 4–5) (*n* = 73)	%
PLDs	47	64.39
Illness	11	15.28
Excessive noise (nightclubs, raves and concerts)	35	47.29
Head, neck and ear injury	37	50.00
Ear-related problems	45	61.81
Medication	11	14.86 (14.86%)

Note: Rating scale: 1 = strongly disagree, 2 = disagree, 3 = unsure, 4 = agree, 5 = strongly agree. Some participants chose more than one option in the questionnaire.

### Awareness and use of hearing protection devices

The majority of 65 (91.78%) participants who answered this question were aware of HPDs. Most of the participants (49.71%) heard about HPDs from their family, while 44.71% had learned about HPDs from the Internet.

**FIGURE 1:**
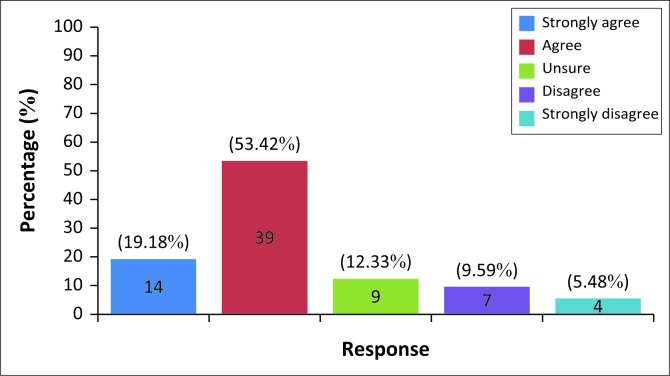
Need of HPDs during exposure to high levels of noise (*N* = 73).

Although the majority, 73.6%, agreed that HPDs should be used when exposed to excessively loud levels of noise, 95.95% stated that they do not wear HPDs when exposed to excessively loud noise (Refer to Figure 1 for participant’s awareness and use of hearing protection devices). Only a small percentage (4.17%) stated that they wore HPDs during excessive noise such as those at nightclubs, raves and concerts. It must be noted that participants were not given any explanation what excessive noise meant. However, as the previous question alluded to the fact that attendance at nightclubs, raves and concerts may cause tinnitus, participants may have understood that to be the meaning of excessive noise in this study.

### Experience of tinnitus

**FIGURE 2:**
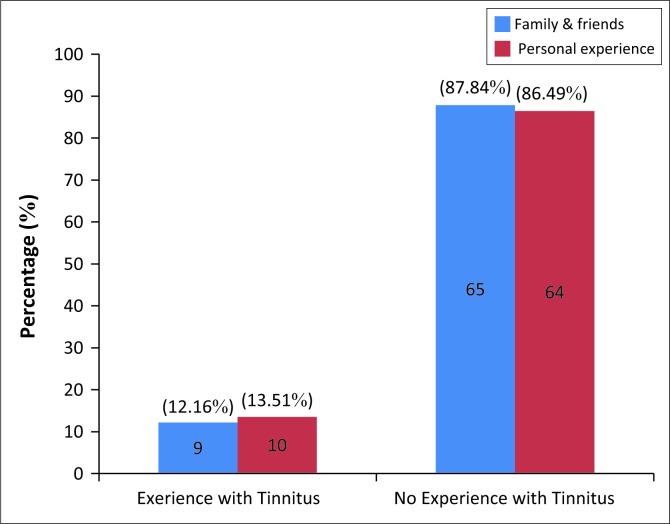
Experience of tinnitus from family and friends and personal experience (*N* = 74).

The results indicated that out of the 74 participants who answered this question, 10 (13.51%) participants had experienced tinnitus themselves and 9 (12.16%) knew of a family member or friend who experienced tinnitus (Refer to Figure 2 for participant’s experience of tinnitus). However, it is clear that the majority had no experience with tinnitus. From the nine participants who indicated that they knew someone who experienced tinnitus, five participants indicated that it was a close family member who had tinnitus, that is, mother, grandmother and grandfather. It was also evident that the tinnitus presented differently to each participant.

### Description of tinnitus experienced

All participants described noticing a ringing in their ears at some point in their lives. Of the 10 (13.51%) participants who personally experienced tinnitus, 40% experienced the sound in both ears, while a small percentage (10%) were unsure of the location of the sound. It was observed that 50% of participants stated a gradual onset of the sound and 50% stated a sudden onset. All the participants (100%) stated that the sound occurred sometimes, which is intermittently. The results also revealed that 50% of participants described the sound as ringing, while one participant described a pulsating sound. According to the results obtained, 90% of participants described the sound as annoying. Approximately a third (30%) said that they found the sound extremely annoying. However, it is not certain whether these participants who found the sound annoying knew what tinnitus actually was.

### Effect of tinnitus on daily life

**FIGURE 3:**
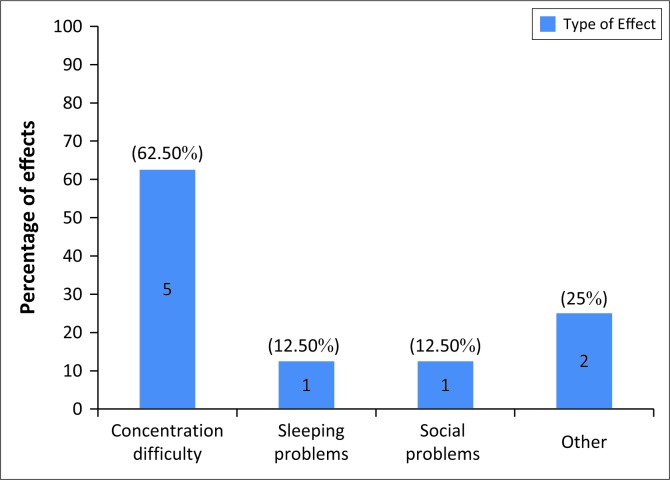
Effects of tinnitus on daily life (*N* = 8).

Only eight of the 10 participants who experienced tinnitus provided information regarding the impact of the tinnitus in their life. Of these, 62.50% complained of concentration difficulties because of the tinnitus. One participant complained of sleeping problems and one participant complained of social problems. However, it must be noted that although only a few participants chose to answer this question, the other participants in this study who did not answer this question or young adults in general with tinnitus may also experience similar effects of tinnitus (Refer to Figure 3 for effect of tinnitus on daily life).

### Management, self-treatment and relief of experienced tinnitus

From the 10 participants who indicated that they had tinnitus, none of them sought any help from a health professional, even those who found the tinnitus bothersome. It is unknown from the survey why they did not seek any help as this question was not included in the questionnaire. Also, none of the participants who had tinnitus experienced any relief with regard to the severity and intrusiveness of the sound. From the 10 participants who reported having tinnitus, 30% said that they had been treating the tinnitus themselves by reducing the levels of noise they are exposed to. It was not clear whether these levels of noise were within the environment or from PLD. The results of this study also revealed that 70% of the 10 participants were doing nothing about their tinnitus. However, only 22% of those three participants who reduced the noise levels were satisfied with the methods they were using to alleviate the tinnitus.

### Request for information on tinnitus

The results of this research indicated that 62% of the participants requested more information about tinnitus. Of these 62% of participants, 58.69% indicated that they would like to know more about the causes and where to seek help for it. It was found that although some of the participants did not know about tinnitus, they still did not request any further information about it.

## Ethical considerations

Ethical clearance was obtained from the Human and Social Sciences Research and Ethics Committee of the University of KwaZulu-Natal to conduct the study (HSS/0497/013M). Once permission was granted by the registrar of the university to access students, a link to the Survey Monkey website was placed on the university’s online notice system, for all students registered in the selected school. This was done with the assistance of the Information Technology Department at the university. Participants could access the information document, consent form, questionnaire and information document via this link. Participants were able to complete a consent form online prior to completing the questionnaire. Only those participants who provided informed consent were able to access the questionnaire. Potential participants had to confirm that they were between 18 years and 30 years and currently registered in the selected school. The consent forms and questionnaires were returned to the researcher electronically once they were completed via Survey Monkey. A reminder was sent to students after 2 weeks to improve the response rate. Data were coded and then analysed with the assistance of a statistician.

## Discussion

### The awareness of the existence of tinnitus

As in the current study, low levels of awareness have also been observed in other studies (Mostafapour et al., 2009). Chung et al. (2005) reported that young adults seemed more concerned about what they perceived as more life-threatening health conditions and as they did not consider complications of the ears as life-threatening, the young adults in their study did not have much concern about tinnitus and hearing loss. However, in this study, it is evident that the majority of participants did not know what the condition was, and therefore, one cannot make an assumption that they may have not considered it life-threatening.

Recent studies have indicate that health promotion programmes regarding tinnitus should be presented to young adults in various ways so that they may be more likely to take advice (Dell & Holmes, 2012; Henderson, Testa & Hartnick, 2010). Also, from a study by Henderson et al. (2010), it was felt to be more beneficial if the source of awareness came from a health professional rather than the Internet. However, it seems that the results from the current study were different. When participants were asked about where they had heard of the condition, a few reported that they knew someone in their family who experienced it and a few came across it while surfing the Internet.

### Awareness of the effects of tinnitus

Literature indicates that the severity of tinnitus is not necessarily a true reflection of the actual impact it has on the individual’s life (Alam et al., 2012). Sometimes, a person can have tinnitus that is mild but may still feel that it has a significant impact on daily life. Also, someone who may have extremely severe tinnitus may feel that it does not impact daily life (McCombe et al., 2001). It is concerning that most participants in this study complained of concentration difficulties. These participants were university students and thus required to concentrate on their studies in order to succeed. These negative effects can drastically prevent young adults from participating in their social contexts and affect their progress academically, socially and vocationally (WHO, 2007). The other negative effects such as reduced social interactions and sleeping difficulties were also reported in this study. These results were similar to those found in a few other studies (Alam et al., 2012; Cho et al., 2013). Concentration difficulty can impact negatively academic performance and it is therefore important to identify this problem earlier in the acquisition of tinnitus. Because the majority of participants in this study indicated that they did not know about the condition, it is even more apparent that education programmes be provided to young adults, highlighting the negative effects of tinnitus. With an increase in awareness of this condition, young adults will be able to recognise it and seek appropriate management, thereby increasing their chances of success in their career and social interactions.

### Awareness of the causes of tinnitus

A study by Gilliver, Beach and Williams (2013) found that there was an urgent need for young adults to be aware of prolonged use of PLDs at high sound levels. Their study concurs with results from other similar studies which found that young adults were not well educated about the causes of tinnitus and as a result, exposed themselves to high levels of noise either from PLDs or from attending nightclubs, concerts and/or raves and recreational activities (Gilles et al., 2013; Dell & Holmes, 2012). However, even after being made aware of the risks, they may still choose to use PLDs because of social reasons (Levey et al., 2012). Perhaps, young adults may only limit the use of PLDs if they have tinnitus or any other ear-related problems after prolonged use. In this study, all the participants who experienced tinnitus stated that it may have been because of noise exposure that was attributed mostly to PLDs. It can be assumed in this study that, perhaps, exposure to noise may have been a contributing cause to the tinnitus.

### Awareness and use of hearing protection devices

The few participants who obtained knowledge about HPDs from family and friends who have used it, as well as the Internet, may, in fact, be knowledgeable about the importance of the use of HPDs. However, they may not be putting this knowledge into practice because of, perhaps, peer pressure and conforming to trends that have been set by their peers. Studies have stated that young adults are likely to behave in a certain way because they want to ‘fit into’ society, that is, in a similar way as their peers (Gilles et al., 2013). It may also be that the participants do not have easy access to HPDs or may not be aware of where to access them; hence, they do not use them. It is unknown whether the participants did, in fact, frequent areas or activities with excessive noise such as nightclubs, concerts, raves and shooting ranges. Although participants may be exposed to places with loud noise such as pubs and malls, it may not warrant the use of HPDs as there is an unlikely chance of acquiring permanent bothersome tinnitus in these noise environments. Mostly, tinnitus can be prevented and understood better if one is aware of its causes. If there are ways in which young adults can protect themselves from acquiring tinnitus, then they need to be aware of those ways. Previous studies have shown that young adults are not aware of HPDs (Gilles et al., 2013; Gilliver et al., 2013). In this study, it became evident that young adults seemed to know about HPDs; however, most of them did not make use of hearing protection when exposed to loud noise. There is no definite reason that can be identified as to why this is so as this question was not included in the survey. Taking precautions when visiting a nightclub or limiting the use of a PLD may be seen as deviating from the social norms and not conforming.

### Experience of tinnitus

Tinnitus may not always be experienced by the individual person but can be experienced via family and friends who may be associated with that person. Therefore, it may be assumed that young adults may be likely to indirectly be exposed to the causes, effects and characteristics of tinnitus from close family and friends who experience it. With regard to the participants themselves having tinnitus, research has shown that young adults are at risk of acquiring tinnitus (Chung et al., 2005). Other recent research by Gilles et al. (2013) also found that tinnitus has increased in the last 20 years in the young population group. One such study found that 89.5% of young adults had transient tinnitus after exposure to excessive noise (Gilles et al., 2013). However, from the results found in this study, one would notice that these results are different from what previous researchers have found. Perhaps, a possible reason could be that this study did not have as many participants as the others. Therefore, these results need to be interpreted with caution.

### Description of tinnitus experienced

According to literature, tinnitus with a gradual onset is generally associated with pathologies such as sensorineural hearing loss and tumours, whereas tinnitus with a sudden onset can result from ear trauma which can be physical or through sudden loud noise exposure (Tyler, 2000). In this study, a few participants felt that noise exposure had caused their tinnitus. Previous studies have also indicated that the onset of tinnitus may be linked to the exposure of excessive noise (Degeest et al., 2014; Landälv et al., 2013). All the participants (100%) who experienced tinnitus stated that it occurs ‘sometimes’. However, it was not requested from the participants in the survey to elaborate on what they meant by ‘sometimes’. Therefore, it is difficult to make further assumptions in this regard. It has been suggested that intermittent tinnitus is related to excessive noise exposure (Gilles et al., 2013). Therefore, it can only be assumed that the participants who complained of hearing tinnitus ‘sometimes’ may have acquired it from exposure to excessive noise. However, if these participants experienced the tinnitus only ‘sometimes’, then the tinnitus may not be as intrusive as it would have been if it were present all the time. This is particularly important for young adults who are most likely involved in academic studies, as permanent tinnitus may negatively affect their studies. Although one must revert to the early results where participants stated that the tinnitus was extremely annoying. This reiterates the fact about the link between the subjective report of tinnitus and the actual objective severity of it.

The results also show that 50% of participants felt that the tinnitus sounds like it is ringing. Ringing tinnitus is usually associated with exposure to loud noise (Holmes & Padgham, 2011), which may allude to the fact that excessive noise exposure may have been the primary cause of tinnitus in those participants who complained of a ringing sound. It must be noted, however, that one participant (10%) complained of pulsating tinnitus. According to the literature, pulsatile tinnitus may be indicative of a possible vascular problem (British Tinnitus Association, 2014). It was not known whether any of the participants suffered from a vascular problem, as there was no specific question in the questionnaire to confirm this information.

### Management, self-treatment and relief of experienced tinnitus

There are various methods that may relieve tinnitus, ranging from counselling to masking and possibly surgery (Canadian Academy of Audiology, 2014). However, none of the participants in this study obtained help from a health professional. It may be assumed that because there was a lack of knowledge with regard to tinnitus, these participants did not know where to go for assistance. Also, as some participants experienced the tinnitus only ‘sometimes’, they may have not felt the urgent need of seeking treatment for it. Because only very few participants (30%) had indicated that they reduced the volume of their music in order to alleviate the problem, this result cannot be generalised to the wider population. One also cannot be sure as to what may have been the actual cause of their tinnitus, which may not have necessarily been excessively loud noise.

## Conclusion

Studies on tinnitus have focused predominantly on the older adult population. However, various studies have established that there is an increase of reported tinnitus in young adults, which motivated the researcher to conduct this study. The study confirmed that a few young adults from this study experienced tinnitus. It must be known that these results cannot be generalised to the wider young adult population because of the small sample size obtained.

It is important that young adults are aware of tinnitus so that they can protect themselves from exposure to its causes and know where to seek help if they experience tinnitus. It needs to be considered that although the Internet is a good online tool to use for health promotion, it may not be accessible to all young adults especially those from a lower socio-economic group. Other studies as those mentioned in the literature above also allude to the fact that a health professional may also be a preferred person to conduct health promotion and awareness on tinnitus.

It was evident that the majority of young adults in this study did not know about tinnitus nor its causes. Health promotion programmes on tinnitus are important as it will ensure that young adults limit their exposure to tinnitus, which may in the long run inadvertently decrease the existence of tinnitus and its negative effects in young adults. Also, by providing knowledge and awareness on the appropriate management for tinnitus, young adults who have tinnitus will know where to go to seek help. This will have a positive effect on their career and overall quality of life.

### Limitations of the study

Although this study has revealed pertinent information regarding tinnitus in young adults, some limitations were identified:

- The expected sample size was not achieved. The sample size was not large enough to make a generalisation to the entire young adult population.
- Detailed questions regarding the participant’s actual exposure to tinnitus, such as attending nightclubs, exposure to illnesses and injury were not included in the questionnaire.
- An internal consistency measure which would have enhanced the reliability of the questionnaire was lacking.
- The use and limitations of electronic surveys in this study were apparent with regard to the low response rate obtained.

### Implications

From the results obtained in this study, the following clinical and theoretical implications are postulated:

- The provision of tinnitus management by health professionals responsible should be more accessible to young adults with tinnitus with regard to their awareness of strategies available and who they can seek help from.
- Audiologists, other health care professionals, young adults and the general public should be more aware about the need for early intervention of tinnitus in young adults to assist with the negative effects of tinnitus.
- Increased awareness of the existence, causes and management of tinnitus may be provided via accessible sources such as the Internet, health talks in the media and health clinics.
- It is important to propose educational programmes at tertiary institutions about hearing loss and associated tinnitus as this is an environment where young adults can be readily accessed.
- There needs to be greater awareness created among young people about the damaging effects of listening to high levels of sound.

### Recommendations for future research

From the study, the following recommendations are suggested:

- A paper-based questionnaire should be used to probe issues like knowledge about tinnitus in young adults. A paper-based questionnaire may provide a better response rate.
- The levels and length of time of noise exposure causing tinnitus in young adults should be further explored.
- More studies need to be conducted on young adults who have tinnitus. These studies should ideally be qualitative studies that focus on the experience of tinnitus, access to management and psychological effects.
